# Fabrication, *in-vitro* characterization, and enhanced *in-vivo* evaluation of carbopol-based nanoemulsion gel of apigenin for UV-induced skin carcinoma

**DOI:** 10.1080/10717544.2017.1344333

**Published:** 2017-07-07

**Authors:** Manmohan S. Jangdey, Anshita Gupta, Swarnlata Saraf

**Affiliations:** University Institute of Pharmacy, Pt. Ravishankar Shukla University, Raipur, India

**Keywords:** Carbopol, apigenin, skin cancer, nanoemulsion, stability, CLSM

## Abstract

The aim of this study was to develop a potential novel formulation of carbopol-based nanoemulsion gel containing apigenin using tamarind gum emulsifier which was having the smallest droplet size, the highest drug content, and a good physical stability for Skin delivery. Apigenin loaded nanoemulsion was prepared by high speed homogenization method and they were characterized with respect to morphology, zeta potential, differential scanning calorimeter study, and penetration studies. *In-vitro* release studies and skin permeation of apigenin loaded nanoemulsion by goat abdominal skin was determined using Franz diffusion cell and confocal laser scanning microscope (CLSM). The cytotoxicity of the reported formulation was evaluated in HaCaT Cells (A) and A431 cells (B) by MTT assay. The nanoemulsion formulation showed droplet size, polydispersity index, and zeta potential of 183.31 nm, 0.532, and 31.9 mV, respectively. The nanoemulsions were characterized by TEM demonstrated spherical droplets and FTIR to ensure the compatibility among its ingredients. CLSM showed uniform fluorescence intensity across the entire depth of skin in nanocarriers treatment, indicating high penetrability of nanoemulsion gel through goatskin. The nanoemulsion gel showed toxicity on melanoma (A341) in a concentration range of 0.4–2.0 mg/ml, but less toxicity toward HaCaT cells. The carbopol-based nanoemulsion gel formulation of apigenin possesses better penetrability across goatskin as compared to marketed formulation. Hence, the study postulates that the novel nanoemulsion gel of apigenin can be proved fruitful for the treatment of skin cancer in near future.

## Introduction

Today, the entire world is increasingly interested in natural medicines and there is a growing Demand for plant-based medicines. Application of novel drug delivery systems with phytoconstituents can lead to enhanced bioavailability, increased solubility and permeability, thereby reducing the dose, and hence, side effects (Kulkarni, 2011). Skin route of administration has been received much attention in the pharmaceutical field because of the more flexibility in designing of dosage form than drug delivery design for others routes. Novel drug delivery provides sustained action at a predetermined rate, constant zero order kinetics, and efficient drug level in the body. The uniform distribution of the multiple unit dosage forms along the skin could results in more reproducible drug absorption and reduced risk of local irritation; this gave birth to local dermal sustained drug delivery and led to development of lipids nanovesicular carriers (Swarnlata et al., 2015; Estanqueiro et al., 2015).

In modern age, with the significant increase in the amount of UV radiation has led to a surge in the increase incidence of skin cancer. The incidence of melanoma increasing at a faster rate than almost all other cancers, cutaneous malignant melanoma is considered to be the most lethal form of skin cancer and is one of the most treatment-refractory malignancies (Mangalathillam et al., 2012; Gupta et al., 2016). Exposure to ultraviolet (UV) radiation is associated with a variety of harmful effects ranging from photoaging to skin cancer or suppress the immune system of the skin. UVB (290–320 nm) directly damages the cellular DNA leading to the formation of the 6–4 cyclobutane pyrimidine dimmers, and UVA (320–400 nm) indirectly damages the DNA via the production of oxygen reaction species (Mukhtar et al., 2005). This dermal therapy has been using natural phytoconstituents or in combination with some chemotherapeutic agents a good option for the better treatment of malignant melanoma (Mangalathillam et al., 2013).

Nanoemulsions have received a growing attention as colloidal drug carriers for pharmaceutical applications (Kanokporn et al., 2010). Nanoemulsions were obtained when the size of an emulsion globule reaches approximately 20–500 nm (Songyot et al., 2012). The small droplet size can resist the physical destabilization caused by gravitational separation, flocculation, and/or coalescence. It has also avoids the creaming process because the droplet’s Brownian motion is enough to overcome the gravitational separation force (Forgiarini et al., 2001; Thadros et al., 2004). Plant polysaccharide has been shown to be useful for the construction of drug delivery systems for specific drug delivery (Malviya et al., 2010). *Tamarindus indica* Linn (*Leguminosa*) is a common and the most important tree of India and South East Asia (Livingston Raja et al., 2008; Jana et al., 2010). Tamarind seed polysaccharide (TSP) has xyloglucan and glucose backbone with xylose and galactose decoration as side chains (Sano et al., 1996; Jana et al., 2010). It is used as binder in tablets, gelling agent, thickening agent, as emulsifier and as stabilizer in food, and pharmaceutical Industries (Kulkarni et al., 1997; Deveswaran et al., 2009).

Apigenin is a polyphenol, non-mutagenic and is capable of selectively inhibiting cell growth and inducing apoptosis in cancer cells without affecting normal cell (Patel et al., 2007). A widely distributed plant flavonoids (4-, 5-, 7-Trihydroxyflavone) present in most of the common fruits and vegetables has been shown to possess anti-inflammatory, anti-carcinogenic effects for skin (Wang et al., 2000; Yin et al., 2001). Apigenin is a strong inhibitor of ornithine decarboxylase, an enzyme that plays a major role in tumor promotion. Apigenin has also been shown to suppress protein kinase cell activity and prevents UVB-induced skin carcinogenesis (Lepley & Pelling, 1997). More importantly, according to previous studies, apigenin could induce apoptosis of cancer cells and exert effects on inhibiting the invasive process (Czyz et al., 2005). However, the solubility of apigenin as a poorly water soluble drug is only 0.0032 mg/ml in water (Zhang et al., 2012) and 0.001–1.63 mg/ml in high hydropholic or nonpolar solvents (Block, 2000), leading to a poor absorption in gastrointestinal tract. Therefore, the improvement in solubility and bioavailability is urgently needed for development and application of apigenin.

Many studies have been published on the production of nanotechnology to incorporate apigenin (Zhai et al., 2013), through their study they demonstrated that the small size (<100 nm) of micelles allows for their efficient accumulation in tumor tissues via the enhanced permeability and retention (EPR) effect. Moreover, polymeric micelle has many other special properties, such as biocompatibility and increased stability. Reports on human carcinoma cells have shown that apigenin induces growth inhibition, cell cycle arrest, and apoptosis (Yin et al., 1999; Wang et al., 2000; Yin et al., 2001). It was also studied to cause selective cell cycle arrest and apoptosis in human prostate carcinoma cells but not in normal cells (Gupta et al., 2001). Although a recent study has shown that apigenin inhibits growth of human hepatoma Hep G2 cells (Yee et al., 2003), information on its anti-tumor properties and cellular mechanism remain limited. So the objective of research work focuses on fabrication and characterization of carbopol based apigenin loaded nanoemulsion gel and its evaluation against UVB irradiation-induced skin cancer malignancies using A431 cell by *in-vitro* methods as well the skin permeation studies to measure the penetration and retention effects.

## Materials and methods

Apigenin was purchased from Sigma Aldrich (Mumbai, India) and Tamarind gum was a gift sample from Hari Om Gum Industries, Surat, Gujarat, India. Castor oil was procured from HiMedia, Mumbai, India and other oils like olive oil, rice bran oil, cotton seed oil, soya oil, and ground nut oil were purchased of analytical grade from Raipur (C.G.). Flonida cream, Shalak Pharmaceutical Ltd., Karampura, New Delhi was purchased from the market. Purified water from ultrapure water system (Synergy UV water purifier system, India) was used throughout the study.

### Method of preparation by high-speed homogenization

Apigenin-loaded nanoemulsions were prepared using high speed homogenization with some modified of a reported method by Bouchemal et al. (2004) and Huang & Yu (2012). Briefly, mixing 10% of oil phase and 90% aqueous emulsifier solution containing 1, 2, 3, 4% w/w of Tamarind gum as emulsifying in ultrapurifier water. Drug dissolved in oil with magnetically stirring at 60 °C for 10 minute, it is oil phase and emulsifying gum dissolved in ultrapurifier water. This was then oil Phase added drop wise in to the aqueous phase with magnetically stirring at 600 rpm. Resulting the Solution mixture were added drop wise in to the Distilled water with mechanically stirring at 1000 rpm and again stirring for 15 min then to formed Coarse emulsion and Coarse emulsion was homogenised at 20,000 rpm for 10 min, for conversion in to nano sized and filtered by 0.22 μ filter paper.

### Formulation of nanoemulsion based gel

Nanoemulsion base gel was prepared as previously described by Modi & Patel (2011), with increased apigenin loading. Briefly, dispersing the 1 g of the Carbopol 934 in a sufficient quantity of distilled water. After complete dispersion, the Carbopol 934 solution was kept in the dark for 24 h for complete swelling. Then the apigenin-loaded nanoemulsion was slowly added to the viscous solution of Carbomer 934 under magnetic stirring. The pH values were subsequently regulated to 5–6. Then other ingredients like isopropyl alcohol (5% wt/wt), PEG-400 (5% wt/wt), PG (5% wt/wt), and triethanolamine (0.5 g) were added to obtain a homogeneous dispersion of gel.

### Screening of oil

The solubility of apigenin were determined in different oils like castor oil, olive oil, cotton seed oil, soya oil, ground nut oil, and rice bran oil, all these oils are easily available and commonly used in nanoemulsion formulations (Pathan & Mallikarjuna Setty 2011). Briefly, the solubility of drug (apigenin) was determined by taking 20 mg of drug in 1 ml of different oils in volumetric flask. The volumetric flasks were tightly stopper and were continuously stirred for 72 h. and then samples were centrifuged at 3000 rpm for 15 min. The supernatant was separated, filtered, and after appropriate dilution with methanol, solubility was determined by UV spectrophotometer at *λ*_max_ 336 nm. The castor oil was selected for the formulation of nanoemulsion because the drug shown highest solubility in castor oil, therefore, the castor oil was selected (Malviya et al., 2009).

### Phytochemical examination of tamarind gum

The phytochemical tests were performed using various reagents. Aqueous solution of extracted gum was used for chemical characterization. Test for carbohydrates, proteins, mucilages, alkaloids, fats, tannins, amino acids, and gums were performed according to standard procedure (Kokate, 1991; Mohamed et al., 2015).

### Morphology

Nano-sized emulsions were selected and investigated by transmission electron microscope (TEM) (Hitachi J 500, H7500 Japan). Shows transmission electron micrographs of nanoemulsions prepared from 1 to 3% (w/w) of tamarind gum in aqueous solution. The TEM images revealed that the oil droplets were spherical and their size was less than 500 nm.

### Droplets size measurement

Droplet size was determined by photon correlation spectroscopy (PCS) that analyzes the fluctuations in light scattering due to Brownian motion of the droplets using a Malvern Zetasizer 3000 HSA (Malvern Instruments, United Kingdom). The formulation (0.1 ml) was dispersed in 50 ml of sufficient amount of ultra-purified water in a volumetric flask and mixed thoroughly with vigorous shaking before conduct of experiment and light scattering was monitored at 25 °C at a 90° angle between laser and detector.

### .X-ray diffraction study

XRD was performed to analyze the nature (crystalline or amorphous nature) of Apigenin loaded nanoemulsion gel. X-ray powder diffraction studies of pure drug Apigenin, Tamarind gum, physical mixture (apigenin and tamarind gum), and nanoemulsion gel were carried out using powder X-ray diffractometer (PANalytical 3 kW X’pert Powder, United Kingdom). Sample were positioned in sample stage and scanned from 2 to 60^θ^ with an operating voltage of 40 kV and current 30 mA.

### FTIR study

The FTIR spectrum (Shimadzu 8400 S) of drug samples (apigenin, tamarind gum, and drug with tamarindus) was used for infrared analysis of samples using KBr pellet technique (Parfitt, 1999). About 1–3 mg of sample was mixed with dry potassium bromide and the samples were examined at transmission mode over wave number range of 4000–400 cm^−1^.

### Zeta potential measurement

Zeta potential for nanoemulsion was determined using Zetasizer (Malvern instrument, Westborough, MA). Samples were placed in clear disposable zeta cells and results were recorded. Nanoemulsions were dispersed in ultra-purified water at the ratio of 1:50 (v/v) and the electric field applied and zeta potential was recorded. The average and standard deviation of the measurement of three batches of emulsions were reported.

### Viscosity measurement

Viscosity is a parameter, which is used to determine rheological properties of any preparation. Viscosity measurements were performed at 25 °C (ambient temperature) using Brookfield Digital Viscometer (Spindle: Low Viscosity-3, Stoughton, MA) at 10, 20, 30, 50, and 100 rpm. The experiment was carried out in triplicate.

### Drug content

The nanoemulsion was suitably diluted (1 ml of nanoemulsion were diluted up to10 ml) with methanol to obtain required drug concentration of 10 μg/ml and absorbance was recorded by using UV spectrophotometer (Perkin Elmer Lambda 25 double beam UV–Visible Spectrophotometer, Singapore) at 336 nm (Pathan & Mallikarjuna Setty, 2011).

### Differential scanning calorimetry (DSC)

DSC is a tool to study the melting and recrystallization behavior of crystalline material like nanoemulsion. DSC examination was performed for the optimized formulation, pure drug and the polymer using a DSC instrument (Mettler 305, Switzerland). Sample of 5 mg were placed in aluminum pans (Al-Crucibles, 40 Al). The probes were heated from 25 to 400 °C and at a heat flow rate of 10 °C/min under nitrogen atmosphere.

### *In-vitro* drug release studies through cellophane membrane

*In-vitro* release studies of drug loaded nanoemulsion and pure drug suspension and marketed product was carried out for each drug individually using modified Franz diffusion (Mol. Wt. cut of 6000–8000, HI Media Ltd., Mumbai, India). Vertical Franz Diffusion cell was designed and validated prior to the release study. The cellophane membrane was mounted on a diffusion cell assembly having diffusion area of 2.5 cm. The receptor compartment consisted of a 22.5 ml phosphate buffer at pH 5.5 as the receptor fluid agitated at 100 rpm, and was maintained at 37 ± 0.5 °C throughout the experiments. The prepared formulation was placed in the membrane of donor compartment (Ali et al., 2012). An aliquot of 2 ml sample was withdrawn at suitable time intervals and replaced immediately with an equal volume of fresh diffusion medium. The % drug release was calculated and graph of % drug release against time was plotted, release studies were performed in triplicate for each formulation (Garga et al., 2015).

### Release kinetics

Based on the results of *in-vitro* drug release studies; graphs were plotted for the models to interpret the kinetic behavior from developed nanoemulsion gel. The models studied were:

Zero order rate kinetics, first order rate kinetics, Higuchi’s kinetics, In this kinetic behavior the graph is plotted between the cumulative percent release with respect to time. Ritger–Peppas exponential kinetics where the graph is between log of cumulative percent drug release with respect to time (Siepmann & Peppas, 2001).

### *Ex-vivo* skin permeation studies

#### Preparation of a goatskin

Fresh goatskin was collected from a local slaughter house. After thorough washing, the hair on the skin was removed using a surgical razor and the skin was separated from the underlying cartilage and subcutaneous fat using a scalpel and cut into appropriate sizes. The skin was stored in formalin solution at 4 °^ ^C. The skin was first immersed in ultra-purified water at 60 °C for 2 min and the epidermis was then peeled off. Dried skin samples were kept under −20 °C for the later use.

#### *Ex-vivo* permeation studies

The full-thickness goatskin was used for the *ex-vivo* permeation experiment using modified fabricated Franz diffusion cell. The skin was clamped between the donor and the receptor chamber of diffusion cell with an effective diffusion area of 2.5 cm^2^. The receptor chamber was filled with freshly prepared phosphate buffer pH 5.5. The diffusion cell was maintained at 37 °C and the solution of the receptor chamber was stirred continuously at 350 rpm by using magnetic stirrer with hot plate (Remi equipments, Mumbai). The formulation (2 ml) was gently placed in the donor chamber at 1, 2, 3, 4, 5, 6, 12, 18, and 24 h, 5.0 ml of the solution in the receptor compartment was removed and analyzed using UV-spectrophotometer (Perkin Elmer Lambda 25 double beam UV–Visible Spectrophotometer, Singapore) and replaced immediately with an equal volume of fresh buffer. All experiments were performed in triplicate (Khan et al., 2015).

#### Skin retention study

The skin retention studies of different formulations were performed in order to analyze the content of apigenin in the skin after 24 h of diffusion. At the end of the experiment, the skin samples were washed up with water and methanol on both sides and carefully dried. After this procedure, a definite amount of methanol was added to each piece of skin. The samples were vortexed for 10 min and stirred overnight. After vortexing, the samples were analyzed by UV spectrophotometer.

#### Skin penetration study of nanoemulsion by CLSM

Skin penetration of Rhodamine 123 was assessed by confocal laser scanning microscopy (CLSM) at Osmania University, Hyderabad, after application to Goat skin *in-vitro* of systems containing probe and nanoemulsion containing, 0.03% rhodamine, were used for the experiments. Nanoemulsion gel was applied homogeneously and nonocclusively to skin. The experiments were run in Franz diffusion cells and the receiver contained phosphate buffer pH 5.5 solution. After 24 h, the skin was removed and washed with phosphate buffer. The skin was then rapidly frozen by liquid nitrogen and a skin surface perpendicular rectangular piece was taken from the site of drug application with the help of a sharp blade. This tissue was fixed on the sample holder with the help of a Tissue frozen medium gel. The skin perpendicular sections (dermis to horny layer) of (250 μm) full thickness were cut with the help of cryomicrotome (Gung, Leica, Germany). The treated area was cut out and tested for probe penetration. The full skin thickness was optically scanned at 15–30 nm increments through the Z-axis of a Leica DMIRE2 confocal laser-scanning microscope (CLSM; Germany) attached to a Leica TCS SP2 fluorescence microscope. Optical excitation was carried out with a 488 nm argon laser-beam and fluorescence emission was detected above 555 nm.

#### Cell culture

All the two cell lines were obtained from the National Center for Cell Science, Pune, India. 3-(4,5-Dimethylthiazol-2-yl)-2,5-diphenyltetrazolium bromide (MTT) was purchased from Sigma Aldrich Ltd.( Mumbai, India), HaCaT cells were cultured in Dulbecco’s Modified Eagle’s Medium Nutrient Mixture F-12 HAM (DMEM F-12 HAM) with 2 mM L-glutamine supplemented with 10% fetal bovine serum (FBS). The cells were incubated in CO_2_ incubator with 5% CO_2_. After reaching confluency, the cells were detached from the flask with trypsin–EDTA. The cell suspension was centrifuged at 3000 rpm for 3 min and then re-suspended in the growth medium for further studies.

#### Cytotoxicity studies

Cytotoxicity experiments were carried out on HaCaT cells and A431 cells lines by MTT assay. MTT [3- (4, 5-dimethylthiazole-2-yl)-2, 5-diphenyl tetrazolium] assay for cytotoxic evaluation is a colorimetric test based on the selective ability of viable cells to reduce the tetrazolium component of MTT to purple-colored formazan crystals (Mangalathillam et al., 2012). Cells were seeded at a cell density of 105 cells/well into 96 well plates and incubated for 24 h at 37 °C in an atmosphere of 95% air and 5% CO_2_. Then, 25 μl of apigenin, plain drug loaded gel and apigenin-loaded nanoemulsion gel, at different concentrations in PBS, was added to culture plates for 24 h. After treatment, cells were rinsed twice with PBS, and the serum free culture medium without phenol red was replaced in all wells. Cells were then incubated for 4 h with the MTT solution (5 mg/ml). The yellow tetrazolium salt was metabolized by viable cells to purple crystals of formazan. The crystals were solubilized overnight in a mixture consisting of 20% sodium dodecyl sulfate (SDS) in HCl (0.01 M). The product was quantified spectrophotometrically by absorbance measurement at a 336 nm wavelength using a microplate reader. The cellular viability was expressed as the percentage of viable cells compared to the control group (Fonseca et al., 2010).

#### Stability studies

Stability studies were tested after 90 d at 4 °C and at ambient temperature 25 °C. The stability studies show that there was a negligible increase in the droplet size from 138.31 ± 0.32 to 138.95 ± 0.73 nm and % creaming rate 100–99.98% during the storage conditions (4 and 25 °C), respectively. The formulation nanoemulsion were showed highest 100% of creaming (low creaming rate) that indicates the formulations are most stable and does not show any phase separation, flocculation. Results revealed that there were no significant changes in the % drug content during storage of formulation for 3 months at 4 and 25 °C. Showed that the viscosity of nanoemulsion was a agreement with the difference at 4 and 25 °C, which is probably due to the presence of emulsifier and its concentration. Zeta potential with slight reduction after 3 months storage at 4 and 25 °C. So, finding nanoemulsion was found to be stable at 4 and 25 °C temperatures for 3 months.

### Statistical analysis

All experiments in this study were repeated at least three times and all the data were expressed as a mean ± SD. The results were statistically analyzed by analysis of variance ANOVA test; *p* values less than .05 (*p* < .05) were considered as significant.

## Results and discussion

### Solubility studies

Solubility studies indicated that apigenin have moderate solubility profile in common solvents. It was freely soluble and better stability in methanol, ethanol, castor oil, and soluble in PBS as compare to other oils and insoluble in distilled water. Significant increase the solubility of apigenin in castor oil and phosphate buffer due to presence of surfactant. The amount of surfactant (sodium lauryl sulfate) was adding by hit and trial method and it was found that 10 ml of 0.5% sodium lauryl sulfate in 1000 ml phosphate buffer responsible for increase the solubility of apigenin. Apigenin in castor oil demonstrates the highest solubility; therefore, castor oil was selected as an internal phase for the formation of nanoemulsions in this study and results are reported in Table 1(A) for solubility of apigenin in different solvents.

**Table 1.** (A) Solubility of apigenin in different solvents; (B) phytochemical examination of tamarind gum; (C) zeta potential of formulations; (D) viscosity of formulations at different rpm.

**(A)**					
Component		Solubility (mg/ml)				
Olive oil		4.45 ± 1.12				
Soya oil		10.63 ± 0.23				
Castor oil		32.24 ± 0.86				
Rice bran oil		6.52 ± 0.28				
Ground nut oil		5.23 ± 0.04				
PBS		16.23 ± 0.97				
Methanol		24.45 ± 1.12				
Ethanol		30.13 ± 0.65				
Water		1.97 ± 0.21				
**(B)**						
S. No.	Tests			Observation		
1	Test for Carbohydrates (Molisch’s test)			+ve		
2	Test for Tannins(Ferric chloride test)			−ve		
3	Test for proteins (Ninhydrin test)			−ve		
4	Test for alkaloids (Wagner’s test)			−ve		
5	Test for glycosides**(**Keller–Killaini test)			−ve		
6	Test for mucilage (Ruthenium red test			+ve		
7	Test for flavonoids (Shinoda test)			−ve		
8	Test for reducing sugar (Fehling’s test)			+ve		
10	Mounting in the iodine (test for starch)			−ve		
14	Test for chlorides (silver nitrate test)			−ve		
15	Test for sulfates (barium chloride test)			−ve		
**(C)**						
Formulations		Zeta potential (mV)				
Pure drug suspension		−26.2 mV				
Nanoemulsion gel		−31.9 mV				
Marketed product		−33.2 mV				
Nanoemulsion		−30.7 mV				
**(D)**						
		Viscosity (cps)				
S. No.	RPM	F1	F2	F3	F4	
1	10	180.23	180.00	180.12	180.10	
2	20	90.17	89.02	89.52	90.23	
3	30	62.04	61.00	60.23	62.42	
4	50	31.23	30.33	30.21	30.42	
5	100	22.61	19.22	19.15	19.21	

Here, F1: Pure drug suspension, F2: Nanoemulsion, F3: Marketed product, F4: Nanoemulsion gel.

### Phytochemical examination of tamarind gum

Phytochemical tests carried out on TSP confirmed the absence of alkaloids, glycosides and tannins. In Molisch’s test, the gum was treated with α-naphthol and concentrated sulfuric acid, which gave violet ring at the junction of two layers. In case of the detection of reducing sugars to the TSP, equal quantity of Fehling’s solution A and B were added. After heating yellow color precipitate was obtained pink color with Ruthenium red and blue color with Benzidine solution indicate the presence of mucilage. To know whether the TSP contains the peroxidase enzyme, which is commonly present in some gums like gum acacia. It was treated with few drops of hydrogen peroxide, no blue color formation; indicate the absence of enzymes in it. Thus a chance of oxidative degradation due to TSP as excipient is eliminated as compared to gum acacia. Mucilage on treating with Ninhydrin reagent does not give purple coloration indicating the absence of amino acids. The results of phytochemical screening of mucilage are summarized in Table 1(B).

### Morphological study

**Figure 1.**
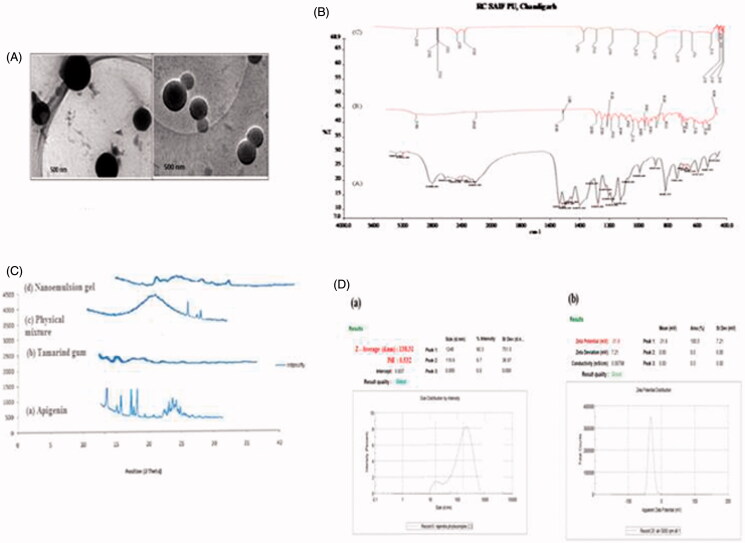
(A) Transmission electron microscope images of apigenin based nanoemulsion; (B) FTIR spectra of (a) apigenin, (b) tamarind gum, and (c) compatibility studies of drug with tamarind gum; (C) X-ray diffractogram of (a) apigenin, (b) tamarind gum, (c) physical mixture, and (d) nanoemulsion gel; (D) (a) mean particle size and polydispersity index of selected batch formulation; (b) zeta potential of the formulation of the selected batch.

The transmission electron micrographs are shown in Figure 1(A). They showed that the prepared vesicles are nano-sized and unilamellar. Outline and core of the well-identified spherical vesicles confirming the vesicular characteristics, displaying the retention of sealed vesicular structure. The vesicles are smaller unilamellar vesicles with a more homogenous size distribution.

### FTIR spectroscopy study

The FTIR spectra of Apigenin showed characteristic peaks at 3550–3200 cm^−1^(O–H stretching), and the C–H stretching occurs above 3000 cm^−1^ with the aliphatic C–H stretching. The very strong bands in FTIR at 3287 cm^−1^ and the C = O stretching bands are observed at in the region 1750–1735 cm^−1^ (C = O stretching). The observed bands at 1651, 1500, 1354, and 1245 cm^−1^ in IR corresponds to ring modes, which are localized on the benzene part of the molecule but disappeared in the nanoemulsion gel formulation, while the new peaks at 1559 cm^−1^ C–C stretching and 1607 cm^−1^ aromatic C–H bending) were appeared. Infrared spectra of drug and polymers were used to study the compatibility between them. No change in peak shows that there was no interaction between drug (apigenin) and tamarind gum and graphically shown in Figure 1(B).

### X-ray diffraction analysis

The X-ray diffraction pattern of Tamarind gum did not show any characteristic peak, which indicates that the structure is completely amorphous. X-ray diffractogram of pure drug Apigenin showed sharp diffraction peak at 2θ value of 13.2, 15.5, 16.6, 18, and 23, tamarind gum displayed characteristic peak at 2θ value of 13.4 and 24.6. The appearance of sharp peak is strongly characteristic of the pure apigenin used in the experiments. As for crystallinity, each samples are amorphous, an indication they are probably easy to be complexation and graphically presented in Figure 1(C).

### Zeta potential measurement

All the formulations were showed lower the zeta potential (higher the negative charges than that of −30 mV), it indicates that increases of a negatively charged polysaccharide (emulsifying gum) in the nano emulsion formulation results in an increase in negative repulsive forces (electrostatic and steric) between oil droplets. Therefore, the presence of emulsifying gum can reduce the interfacial tension and formed a cohesive interfacial film around the emulsion droplets thereby retarding emulsion instability). So all formulations were found to be stable and results are reported in Table 1(C) and graphically shown in Figure 1(D).

### Viscosity measurement

By determination of viscosity of nanoemulsions it was found that viscosity of emulsion decreases with increasing speed of spindle or rate of shear. Viscosity was depends on the concentration of emulsifier in nanoemulsion. So in this manner, the prepared emulsion follows Newtonian law of flow. Results are shown in Table 1(D).

### Effect of tamarind gum concentration

Increasing of tamarind gum concentration in the formulation tended to decrease zeta potential of emulsions. It is thought that the increase of a negatively charged polysaccharide like tamarind in the emulsion formulation results in an increase in negative repulsive forces (electrostatic and steric) between oil droplets. Therefore, the presence of tamarind can reduce the interfacial tension and form a cohesive interfacial film around the emulsion droplets thereby retarding emulsion instability (Funami et al., 2007). Higher concentrations of tamarind gum produced more stable emulsions in which tamarind was tightly bound onto the oil droplets.

### DSC study

**Figure 2.**
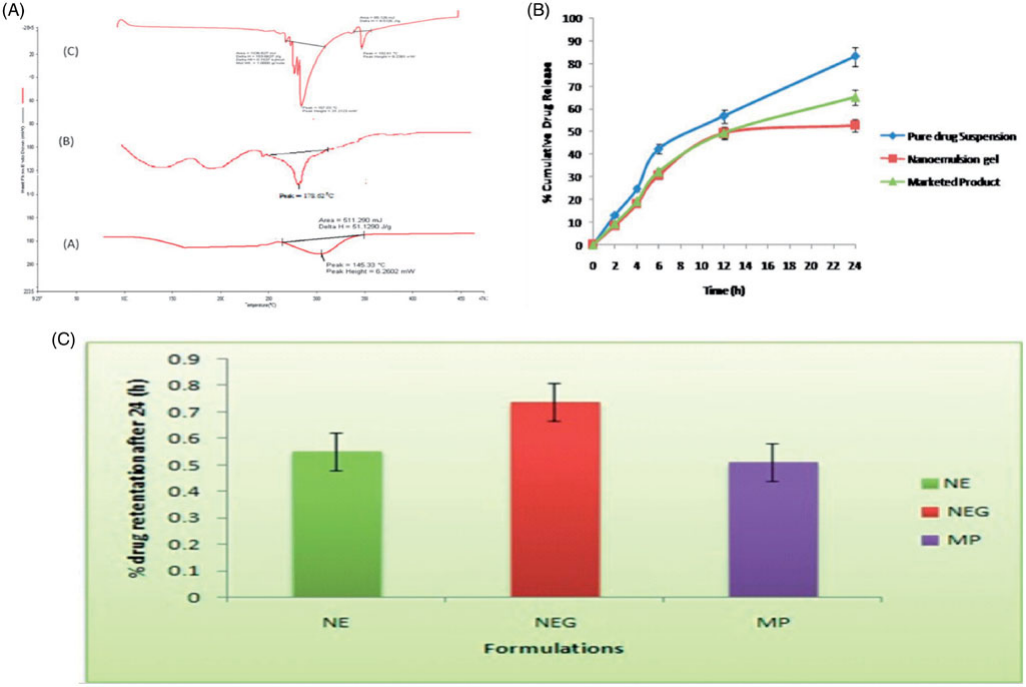
(A) DSC Thermogram of (a) apigenin, (b) drug loaded formulation, and (c) marketed product; (B) *In-vitro* drug release profile of apigenin-loaded nanoemulsion gel and marketed and pure drug suspension in skin pH 5.5; (C) percentage drug retention of different formulation in goatskin after 24 (h).

The DSC scan for Apigenin, marketed product and optimized formulation are shown in Figure 2(A). A sharp melting transition of pure drug was observed at 226.98 °C. The marketed product showed an endothermic peak at 178.62 °C. A DSC thermo gram of formulation showed the peak at 145.33 °C. Thermogram shows more reduction in size and intensity of the endothermic peak of the drug which may be due to its solubility in the nanoemulsion ingredients.

## *In-vitro* release studies

The percent cumulative drug release of apigenin loaded nanoemulsion gel, pure drug suspension and marketed product were investigated by *in-vitro* over a period of 24 h using a cellophane membrane. Results revealed that the 3% emulsifying gum with nanoemulsion gel had the lowest amount of drug release almost 52.90 ± 0.95 was observed after 24 h as compared to other pure drug suspension and marketed product dispersion was 83.24 ± 0.65 to 65.24 ± 1.54, respectively. The outcomes exhibit that the formulation represents burst release phase corresponding to about 10–15% was observed within 2 h due to the drug deposition and release from the nanoemulsion surface. But after 2 h release of the drug from nanoemulsion gel, there was retardation that represent the sustained release pattern. The findings of *in-vitro* release suggested the burst release of drug was due to availability of the free apigenin in the outer surface of the nanoemulsion (Mukerje & Vishwanatha 2009). The sustained release of the drug was due to apigenin and could be the reason for the sustained release of the drug from the internal lipid phase after the initial burst release. The results of drug release are reported in Table 2(A) and Figure 2(B).

**Table 2.** (A)* In-vitro* drug release profile of apigenin-loaded nanoemulsion gel and marketed and pure drug suspension in skin Ph 5.5. (B) Release behavior of Apigenin from Nanoemulsion Gel formulations; (C) Permeation and % drug retention data for apigenin loaded nanoemulsion gel, marketed and pure drug suspension across abdominal goatskin.

**(A)**						
		% Cumulative drug release from different formulations						
S. No.	Time interval	Pure drug suspension	Nanoemulsion gel	Marketed product				
1	0	0	0	0				
2	2	12.90 ± 0.56	8.42 ± 2.35	9.35 ± 1.08				
3	4	24.67 ± 1.98	18.03 ± 0.65	19.02 ± 0.54				
4	6	42.43 ± 2.04	30.75 ± 2.87	32.12 ± 0.24				
5	12	56.78 ± 1.34	40.25 ± 1.43	49.58 ± 2.12				
6	24	83.24 ± 0.65	52.90 ± 0.95	65.24 ± 1.54				
**(B)**								
Formulation	First order		Higuchi		Ritger–Peppas				
Nanoemulsion gel	*K*	*R* ^2^	*K*	*R* ^2^	*n*	*R* ^2^			
	0.072	.939	0.304	.986	0.642	.955			
Marketed product	0.58	.98	0.68	1.02	0.84	1.05			
Pure drug suspension	0.67	.99	0.74	1.28	0.96	1.24			
**(C)**									
Formulation	Jss (μg/ch^−2^/h)	*P* (Ch/h)	LT (h)	D^d^ (Ch^2^/h)	% drug retention after 24 h				
Pure drug suspension	5.43 ± 1.02	0.249 ± 1.42	2.4	6.5	0.55 ± 0.23				
Nanoemulsion gel	6.68 ± 0.46	0.235 ± 1.56	1.8	8.6	0.74 ± 0.05				
Marketed product	5.60 ± 0.63	0.224 ± 1.59	2.0	7.0	0.51 ± 0.30				

Where, K: Release rate constant; *R*^2^: coefficient of determination and *n*: release exponent.

Where, Jss: transdermal flux, *P*: permeability coefficient, LT: lag time, D^d^: diffusion coefficient

### Release kinetics analysis

Release kinetics from the apigenin loaded nanoemulsion gel formulation was compared to pure drug suspension and marketed product from different kinetic models reported in Table 2(B). Results showed that the model was best fitted with data in the Higuchian equation (*R*^2^ = .986), the value of coefficient of determination was about again > .9. Sustained drug release was observed for apigenin during the entire period of the study showed a delayed burst release. The *R*^2^ value of first order for apigenin loaded with nanoemulsion gel were near to one, thus; release of drug apigenin from nanoemulsion gel formulations followed first order release kinetic with *R*^2^ = .955 in Ritger–Peppas equation. The drug release from the presently developed nano system is mostly by the diffusion and is best described by Fickian diffusion. But in case of possible swelling in the system, other processes in addition to diffusion play an important role in exploring the drug release mechanisms. The drug release from this system is described by Fick’s second law of diffusion (Siepmann & Peppas, 2001).

### *Ex-vivo* skin permeation and skin deposition study

The cumulative amount of apigenin permeated through the abdominal goat skin in all vesicle formulations was significantly (*p* < .05) higher than the emulsion and marketed product. The amount of apigenin permeated through skin from Nanoemulsion gel was significantly (*p* < .05) higher than that from pure drug suspension and marketed product. Flux of Nanoemulsion gel through goat skin was 6.68 ± 0.46 and % drug retention was 0.74 ± 0.05, while pure drug suspension and marketed product were significantly lower transdermal flux and % drug retention. The nanosize as well as the influence of surfactant is the major factors for contributing to better the penetration. So surfactant swells the stratum corneum and the intact vesicle can penetrate into and through the intact skin. The results of drug release are reported in Table 2(C) and graphically shown in Figure 2(C). Values represent as mean ± SD (*n* = 3).

### Confocal laser scanning microscopic study

**Figure 3.**
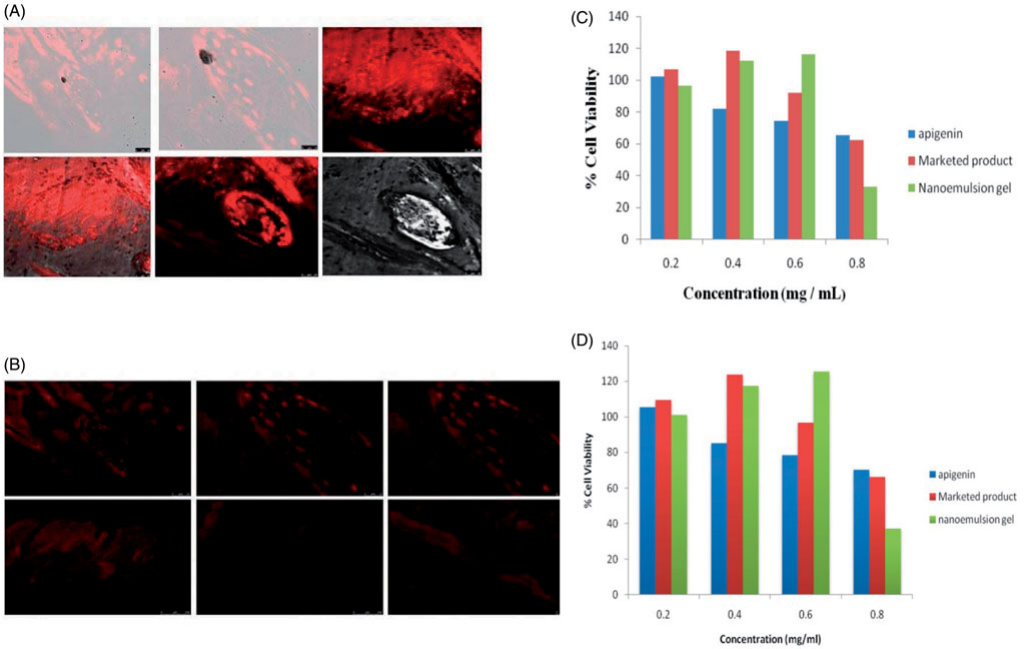
(A) The confocal image showing the depth of skin penetration of Rhodamine red (florescence marker) from nanoemulsion into the goatskin after 24 h. (B) CLSM Photomicrograph of goatskin after 24 h application florescence probe Rhodamine red shows the deposition florescence probe into the skin when applied in the form of marketed product. (C,D) The results of MTT assay on HaCaT Cells (C) and A431 cells (D).

CLSM is widely used in studying vesicle-mediated skin transport of drug molecules (Sarwa et al., 2013). The localization of a permeating molecule in skin layers is made possible with a fluorescence study or radiotracer and visualization of images parallel to the surface of the sample, at multiple depths without mechanical sectioning of the sample is a major advantage of this sophisticate instrument. Confocal laser scanning microscopic study was carried out for both nanoemulsion gel and marketed (Flonida cream, Shalak Pharmaceutical Ltd., Karampura, New Delhi) formulations. Representative images are shown in Figure 3(A,B)). For nanoemulsion gel, uniform fluorescence intensity was observed across the entire depth of skin, extending up to the last section of the examined skin. In contrast, penetrability of nanoemulsion was limited to the 6th section (upper epidermis layer) of skin as intact nanoemulsion was visualized in CLSM. Kirjavainen et al. (1999) have also demonstrated that fluorescent liposome did not penetrate into the skin when delivered from aqueous solution but penetrated into the deeper region from ethanolic solution. Zhang et al. (2011) have studied the nanoemulsions developed in these work likely exerted similar effects as liposomes. However, it is apparent that the intact nanoemulsion gel was retained in the treated skin leading to comparatively lesser penetration into deeper tissues than marketed product probably due to the rigid nature of nanoemulsion gel. Moreover, low-fluorescence intensity was recorded with nanoemulsion as compared to nanoemulsion gel, which reveals that lower the cumulative amount of drug was permeated from nanoemulsion.

### Cytotoxicity studies

The effect on cell proliferation of apigenin as well as the marketed product and apigenin-loaded nanoemulsion gel was evaluated. The incubation with apigenin for 24 h induced a concentration-dependent inhibition of cell proliferation as determined by MTT assay. For this study, the amount of apigenin present in apigenin loaded nanoemulsion gel was equivalent to 0.4892/5 mg. The inhibition was 18.28, 23.34, and 32.50% at 100, 200, and 400 μg/ml of apigenin, respectively. The evaluation of unloaded and apigenin-loaded nanoemulsion gel cytotoxicity demonstrated that, until the concentration corresponded to 200 μg/ml of apigenin, the formulations protected against apigenin cytotoxicity since no inhibition of cell proliferation was observed. Nevertheless, for the highest concentration studied, the reduction in cell viability was about 60% for drug loaded nanoemulsion gel, due to the sum of the cytotoxic effects of both apigenin and marketed product. Results are shown in Table 3(A,B)) and images are shown in Figure 3(C,D)).

**Table 3.** (A,B) Effect of apigenin, unloaded and apigenin-loaded nanoemulsion gel on cellular viability (as % of control) of MTT assay on HaCaT Cells (A) and A431 cells (B).; (C) Stability studies of the formulations at different days and temp conditions.

**(A)**					
	Cellular viability (% of control)				
Apigenin concentration (μg/ml)	Apigenin	Marketed product	Drug loaded nanoemulsion gel		
50	102.34 ± 5.62	106.74 ± 5.18	96.43 ± 7.64		
100	82.24 ± 8.55	118.43 ± 8.40	112.16 ± 8.27		
200	74.56 ± 12.62	92.21 ± 15.45	116.37 ± 8.35		
400	65.52 ± 4.32	62.55 ± 23.48	33.32 ± 10.73		
**(B)**					
	Cellular viability (% of control)				
Apigenin concentration (μg/ml)	Apigenin	Marketed product	Drug loaded nanoemulsion gel		
50	105.54 ± 6.03	109.66 ± 5.16	101.21 ± 6.65		
100	85.11 ± 9.42	123.73 ± 8.4	117.49 ± 5.24		
200	78.52 ± 14.87	96.87 ± 12.46	125.62 ± 6.33		
400	70.32 ± 7.54	66.35 ± 20.32	36.97 ± 09.56		
**(C)**					
Storage condition	% Creaming	Droplet size(nm)	Drug content (%)	Viscosity (cps)	ZP (−mV)
Day 0	100	138.31 ± 0.32	98.23%	62.42	−31.9
Days 30 at 4 °C	100	138.20 ± 0.11	98.14%	62.40	−31.8
Days 30 at 25 °C	99.21	138.17 ± 0.15	98.16%	62. 36	−31.02
Days 90 at 4 °C	99.30	138.42 ± 0.62	97.61%	62.54	−32.24
Days 90 at, 25 °C ambient temperature	99.98	138.95 ± 0.73	98.05%	62.08	−30.56

Results are represented by means ± SD (*n* = 6).

Results are represented by means ± SD (*n* = 6).

### Stability test

The stability of nanoemulsions was studied. Freshly prepared emulsions were milky white in color and showed 100% cream in all preparations. For the tamarind gum polymeric based emulsions, after 60-d storage, the size of oil droplets was no significant changed to when kept at the ambient temperature (25 °C) and at 4 °C (in a refrigerator). Different concentration of tamarind gum influence the stability of emulsions in both conditions, according to the size of oil droplets and percent creaming. However, it was influenced by the concentration of tamarind. Using at least 2% (w/w) of tamarind gum could produce the stable oil-in-water emulsions for 7 d when kept at 4 °C. Burapapadh et al. (2010) studied that after the stability test the emulsions with apigenin provided more stable emulsions with low creaming rate in using higher concentration of tamarind. It appears that tamarind at a high concentration is efficient for stabilization of the tamarind gum based emulsions, i.e. the formulations using 3% (w/w) tamarind showed the highest percent creaming (100% cream) when kept at 4 °C or at ambient temperature (25 °C). The data are represented in Table 3(C).

## Conclusion

In this research work, the carbopol-based apigenin loaded nanoemulsion gel was formulated by high-speed homogenization method and evaluated against skin cancer using two different cell lines. The oil in water nanoemulsion were formulated of the drug apigenin which show good bioavailability because of better solubility in lipophilic solvent, and it can solubilized high amount of castor oil which is commonly used for the nanoemulsion formulation than oil phase, homogenizing in aqueous phase by using emulsifying gum. Apigenin-loaded nanoemulsion formulations were in nanorange 100–500 and showed good physical stability and lack of phase separation or precipitations. The formulations having 3% w/w of emulsifying gum showed highly stable and their size range is 138.31 nm. The drug release study exhibited sustained drug action from the nanoemulsion following higuchi release kinetics (*R*^2^ = .978) as compared with pure drug suspension and marketed product first order kinetics (*R*^2^ = .99 and *R*^2^ = .98). CLSM showed uniform fluorescence intensity across the entire depth of skin in nanocarrier treatment, indicating high penetrability of nanoemulsion gel through goat skin and enhances retention of drug through skin. Apigenin loaded nanoemulsion gel showed reduced cytotoxicity on A431 cells as compared to HaCaT cell lines. The nanoemulsion gel formulation of apigenin possesses better penetrability across goatskin as compared to marketed formulation. Stability study confirmed that nanoemulsion were stable for 3 months at a temperature range of 4 and 25 °C. Furthermore, *ex-vivo* skin permeation showed presence of significant amounts of apigenin into the melanocytes layers of skin. Hence, it can be concluded that novel formulation of apigenin can be proved fruitful for the treatment of skin cancer in near future.
